# Unexpected Diagnosis of Fahr’s Disease in a Patient with Severe Obesity and a Heterozygotic Variant in the *TMEM67* Gene

**DOI:** 10.3390/genes16121406

**Published:** 2025-11-26

**Authors:** Katarzyna Piekarska, Paulina Oczoś, Julia Grzybowska-Adamowicz, Ewa Zmysłowska-Polakowska, Michał Pietrusiński, Agnieszka Zmysłowska

**Affiliations:** 1 Department of Clinical Genetics, Medical University of Lodz, Pomorska Str. 251, 92-213 Lodz, Poland; katarzyna.piekarska2@student.umed.lodz.pl (K.P.); julia.grzybowska-adamowicz@umed.lodz.pl (J.G.-A.); michal.pietrusinski@umed.lodz.pl (M.P.); 2 Department of Radiology and Diagnostic Imagining, Medical University of Lodz, 92-213 Lodz, Poland; paulina.oczos@umed.lodz.pl; 3 Department of Endodontics, Medical University of Lodz, 92-213 Lodz, Poland; ewa.zmyslowska-polakowska@umed.lodz.pl

**Keywords:** Bardet–Biedl syndrome, Fahr’s disease, obesity, heterozygous carrier

## Abstract

**Objective**: The genetic causes of obesity are complex and include diabetes and obesity monogenic syndromes like autosomal recessive Bardet–Biedl syndrome (BBS). Other clinical manifestations of this syndrome include metabolic disorders, polydactyly, retinal dystrophy, and endocrine, urological, and neurological abnormalities. Moreover, isolated clinical manifestations have been described in carriers of heterozygous mutations in BBS genes. On the other hand, Fahr’s disease is characterized by the accumulation of calcium deposits in various areas within the brain, leading to neurodegeneration, and the course of the disease is variable. **Case presentation**: We present the case of a 21-year-old female with severe obesity, diagnosed at the age of six years. The patient also experienced hypertension, hyperlipidemia, insulin resistance, and polycystic ovarian syndrome. During an MRI examination, hyperintensity in the region of the dentate nuclei and hyperintensity in the globus pallidus were described. NGS (next-generation sequencing) results showed a heterozygous variant in the *TMEM67* gene, which revealed the patient to be a carrier of BBS, and a homozygotic variant in the *MYORG* gene, leading to a Fahr’s disease diagnosis. However, due to an insufficient number of phenotypic criteria and only one causative variant in the *TMEM67* gene, the diagnosis of BBS could not be established. **Conclusions**: Attempts to identify the cause of obesity can lead to unexpected results, which can be resolved through collaboration between clinicians of different specialties and the use of NGS molecular testing. The status of being a BBS carrier, which coexists with Fahr’s disease, may be a potential contributing factor to severe obesity and metabolic disorders in the patient.

## 1. Introduction

Bardet–Biedl Syndrome (BBS) is a rare genetic ciliopathy inherited autosomal recessively. Typical symptoms include early childhood obesity, which may be accompanied by insulin resistance (with acanthosis nigricans), hypertension, hypertriglyceridemia, and type 2 diabetes, as well as retinal dystrophy and other ophthalmic abnormalities [1]. To make a clinical diagnosis, a patient must meet either four of the major features or three major plus two minor features. Major features include rod–cone dystrophy, polydactyly, hypogonadism, renal anomalies or dysfunction, impairment of cognitive abilities, and obesity. Among the minor features, there are speech delay, developmental delay, genitourinary abnormalities, dental irregularities, brachydactyly, syndactyly, anosmia or hyposmia, ataxia, congenital heart disease, and type 2 diabetes [2,3]. Patients typically report hyperphagia caused by dysregulation of mechanisms in the central nervous system, leading to fat tissue accumulation [4]. Some of the causative mutations that underlie BBS are also thought to play a significant role in odontogenesis, which may also have clinical implications in these patients [5,6,7]. The life expectancy of patients with BBS may vary depending on the type and severity of symptoms. Most premature deaths are caused by renal impairment [4].

There is no causal treatment available for BBS patients; only symptomatic treatment is used. Polydactyly is surgically corrected [8]. Obesity can be initially treated by non-pharmacological methods such as diet and physical activity. Furthermore, recent studies in mice have shown that a peptide similar to glucagon-like peptide-1 (GLP-1) may be effective in reducing excess body fat in patients with BBS [8]. In addition, setmelanotide has also been approved by the FDA (Food and Drug Administration) and EMA (European Medicines Agency) for the treatment of obesity in patients with BBS through reduction in hyperphagia [9,10]. More invasive interventions for patients include bariatric surgery [1]. To date, it has been established that more than 20 different genes are associated with BBS [2,3]. However, this complex molecular diagnosis, currently provided using the NGS (next-generation sequencing) method in combination with known primary and secondary clinical criteria, allows for a precise diagnosis of BBS [2,11].

On the other hand, Fahr’s disease is a heterogenic group of disorders affecting calcium metabolism, which causes abnormal storage in multiple regions of the brain, especially in the basal ganglia. Some types of this condition may be caused by genetic factors. It affects less than 1 in 1,000,000 individuals [12]. To date, seven genes have been linked with Fahr’s disease, with both dominant and recessive inheritance patterns [13]. Although first symptoms usually occur before the age of 40, pathologies may be seen in diagnostic imaging up to 20 years prior [14]. Initial manifestations include fatigue, impaired coordination, muscle spasms, involuntary movements, dysphagia, and dysarthria. As the disease progresses, motor disorders become more apparent, resulting in tremors, Parkinsonism, and dyskinesias. Neuropsychiatric symptoms may include concentration and memory impairment, changes in behavior, or dementia. Some patients experience headache and vertigo, urinary incontinence, and epileptic seizures [13]. Some reports also mention dental abnormalities such as impacted teeth, delayed eruption of teeth, or hypodontia [15]. Life expectancy varies depending on the case—some patients may live as long as healthy individuals [16]. Symptoms management focuses on pharmacotherapy, including analgesics, anticholinergics, selective serotonin reuptake inhibitors, neuroleptics, and antileptics. Physical rehabilitation is also recommended to maintain satisfactory mobility [16].

The case of a 21-year-old female with genetic variants responsible for both diseases is presented below.

## 2. Case Presentation

### 2.1. Detailed Case Description

A 21-year-old female was referred to the Rare Diseases Outpatient Clinic for Children and Adolescents and Diabetogenetics with suspected monogenic obesity.

The patient was born at 37 weeks’ gestation with a birth weight of 3600 g and an Apgar score of 10/10. In early childhood, the patient experienced rapid weight gain. At 6 years of age, she was diagnosed with Class III obesity. That same year, she was admitted to hospital due to recurrent headaches, leading to diagnoses of hypertension (125/80 mmHg) and hyperlipidaemia. At the age of 7, an impaired fasting glycaemia (IFG) diagnosis was made following an oral glucose tolerance test (OGTT), along with insulin resistance (HOMA-IR 11.22). The patient was treated with metformin and bisoprolol. In addition, magnetic resonance imaging (MRI) of the central nervous system showed no abnormalities. At 9 years of age, an abdominal ultrasound revealed liver steatosis. One year later, due to continuously increasing cholesterol levels (total cholesterol 283 mg/dL; triglycerides 274 mg/dL; LDL-cholesterol 188 mg/dL), treatment with fenofibrate was initiated. At the time, glycated hemoglobin (HbA1c) was 5.4% with HOMA-IR 26.

**Figure 1:**
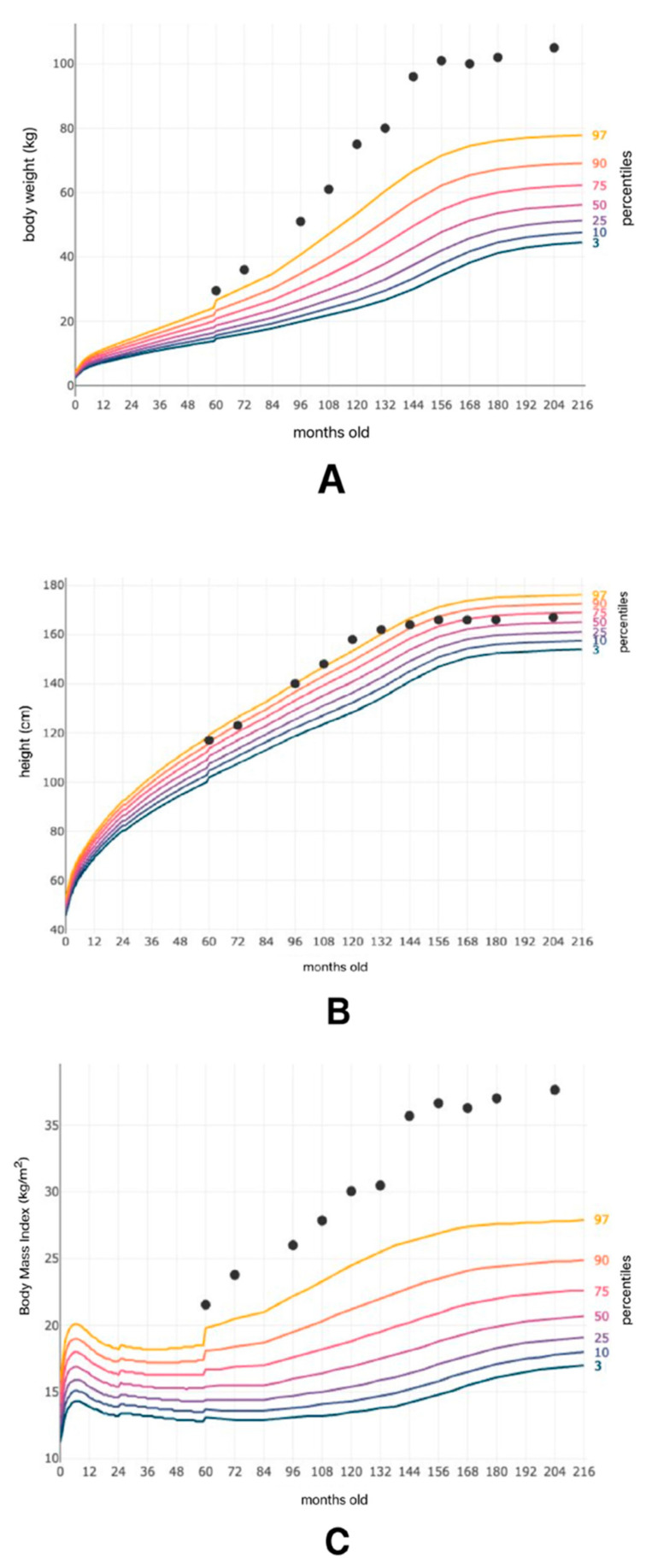
Patient’s weight (**A**), height (**B**), and Body Mass Index (**C**) in relation to the patient’s age in months. Percentiles are marked as color lines.

At 11 years of age, an ultrasound revealed multiple ovarian follicles, and the patient was diagnosed with polycystic ovarian syndrome (PCOS). Additionally, acanthosis nigricans skin lesions were noticed. During hospitalization at the endocrinology department, Cushing syndrome, congenital adrenal hyperplasia, and hypothyroidism were excluded. Two years later, due to inadequate blood pressure control, the antihypertensive treatment was intensified (valsartan, nebivolol, and indapamide). At the age of 17, the first signs of hypertensive retinopathy were observed. Additionally, the patient had learning difficulties, as well as memory and concentration disorders. Her weight continued to increase over the years, reaching 107 kg during the COVID-19 pandemic. Currently, her BMI is 37.7 kg/m^2^. Changes in the patient’s weight, height, and BMI are presented in Figure 1. Her calcium and phosphate metabolism has not raised any concerns so far (calcium level: 9.5 mg/dL; parathormone level: 10.6 pg/mL), except for a reduced vitamin D3 (25-OH) level of only 17 ng/mL.

In the family history, the patient’s mother was diagnosed with obesity, short stature, hypertension, and hypertriglyceridemia. The patient’s father was overweight, with hyperglycaemia and hyperlipidaemia. Written informed consent for genetic and scientific research was obtained from the patient and her parents.

### 2.2. Molecular Analysis

Molecular testing by NGS method revealed the patient to have a heterozygous variant NM_153704.6: c.1379G>C in *TMEM67*, which was located in exon 13, leading to the conversion of arginine to threonine at position 460 of the polypeptide chain. The variant has been described in the ClinVar and dbSNP databases and is classified as a variant of unknown significance (VUS) according to the American College of Medical Genetics and Genomics (ACMG) guidelines. The analysis of variants within the family found the mother, who also presents with obesity, to be a carrier of this variant.

**Figure 2:**
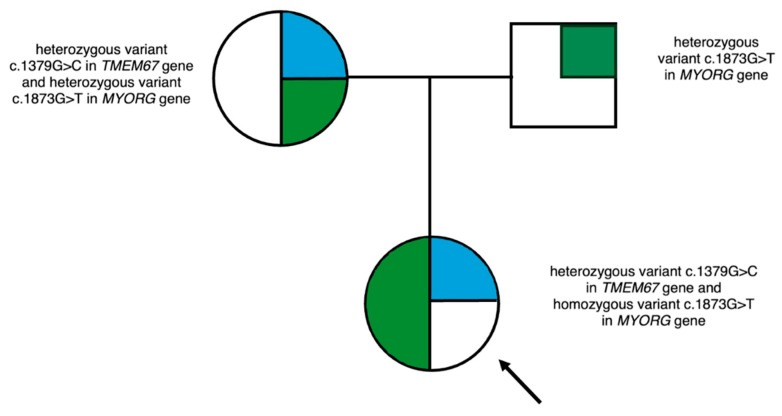
Family Pedigree. Mutation in the *TMEM67* gene (blue), mutation in the *MYORG* gene (green).

The molecular analysis also identified a homozygous variant in the *MYORG* gene: NM_020702.3: c.1873G>T. The variant was located in exon 2, leading to the conversion of glutamic acid to stop codon at position 625 and the premature termination of the polypeptide chain. The latter variant has been described in the ClinVar and dbSNP databases and is classified as pathogenic according to ACMG recommendations. Analysis of variants within the family revealed both parents to be carriers of a heterozygous variant in the *MYORG* gene. The family pedigree is shown in Figure 2.

### 2.3. Imaging

**Figure 3:**
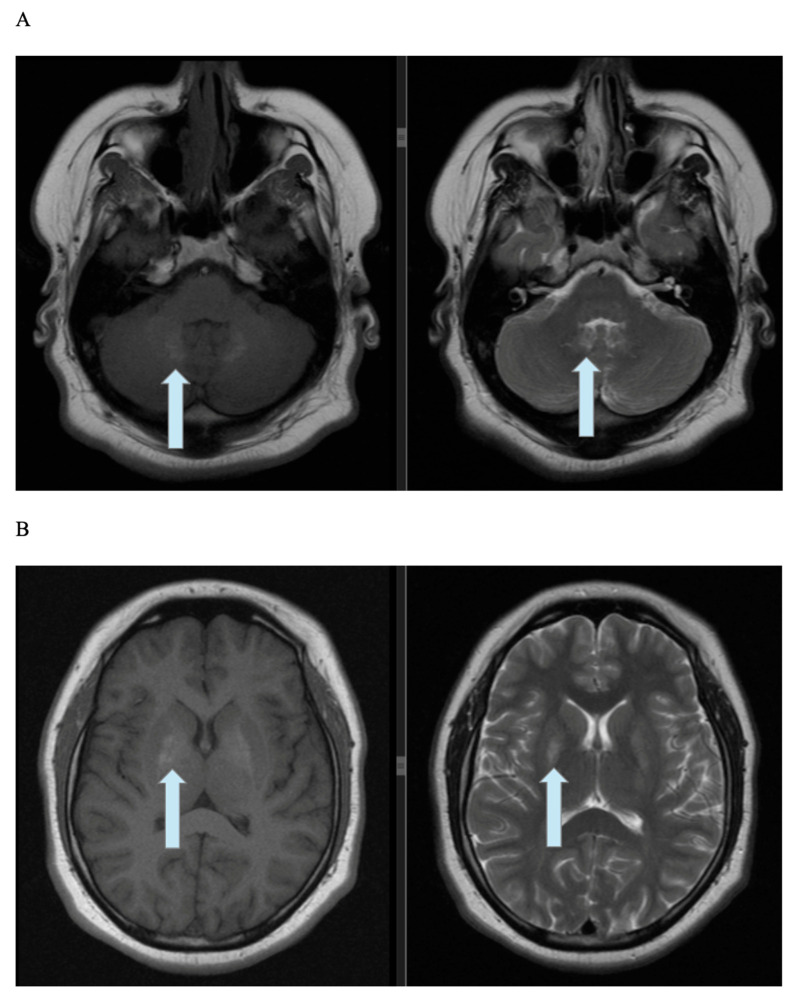
(**A**) T1WI and T2WI MRI images showing symmetrical hyperintensity in the region of the dentate nuclei. (**B**) T1WI and T2WI MRI images showing bilateral hyperintensity in the patient’s globus pallidus.

The patient’s brain MRI (Figure 3A,B) demonstrated symmetrical, poorly separated, and quite intensive hyperintensity within the globus pallidus on both T1-weighted (T1WI) and T2-weighted (T2WI) images. Additional findings included a 4 mm area of moderately elevated signal intensity in the right thalamus and similar hyperintense areas in the region of the dentate nuclei. No other changes in white matter or gray matter were noticed. The signal was normal and ordinary. There were no regions of water-restricted diffusion or pathological contrast enhancement in the cerebrum. The corpus callosum was of typical, standard size without any focal changes; the subarachnoid space was of normal width, and the ventricular system was unwidened.

### 2.4. Dental Examination

**Figure 4:**
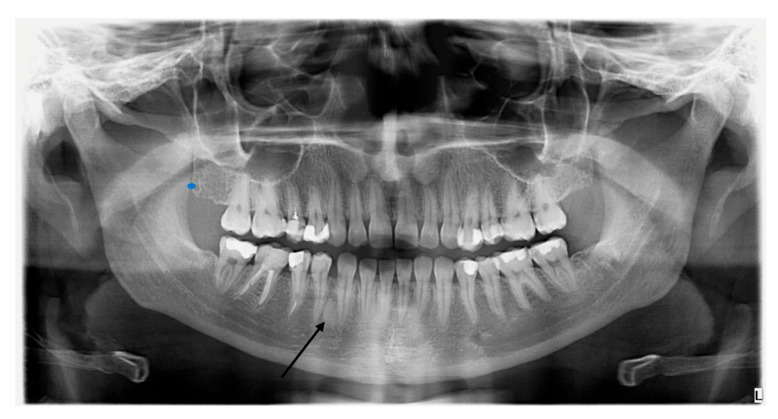
Pantomographic image of the patient showing an asymmetrical lesion in the mandible.

Due to the possibility of dental disorders occurring in both BBS and Fahr’s disease, the patient underwent an intraoral examination supplemented with a pantomographic image. Dental examination excluded periodontal disease and other abnormalities. The pantomogram revealed an asymmetric lesion in the mandible, which, after differentiation from a possible calcium accumulation, was finally classified as a bone island (Figure 4). The lesion is located within the cancellous bone of the mandible between the medial surface of the root of tooth 43 and the distal surface of the root of tooth 44 (canine–premolar). However, this requires further monitoring of bone changes in the area.

## 3. Discussion

No variants were found in the patient’s genes associated with monogenic obesity or genetically determined insulin resistance. However, the presence of variants in two genes caused diagnostic difficulties. First, the patient may be considered a carrier of Bardet–Biedl syndrome, as she does not meet other major criteria for BBS, apart from the presence of one segregating variant in the family. Next, the homozygous, pathogenic variant in the *MYORG* gene leads to the diagnosis of Fahr’s disease. The 21-year-old patient most likely has not presented clinical features of this disorder yet, which matches the course of the illness. The diagnosis was based on changes observed on the MRI. While the patient’s neurological symptoms were initially attributed to her BBS carrier status, in rare cases, symptoms of Fahr’s disease have been described as early as the first decade of life [17].

It is now known that Fahr’s disease is a rare genetic neurodegenerative disorder that results in extensive bilateral cerebral calcifications. Idiopathic calcifications with cognitive and neurobehavioral manifestations are components of Fahr’s disease, which should be differentiated from Fahr’s syndrome, which has a similar MRI picture but develops secondary to an underlying disorder (e.g., hypoparathyroidism, hypothyroidism) [18,19]. In Fahr’s disease, the globus pallidus is most commonly affected, followed by the putamen, caudate, and thalamus. Additionally, involvement of the cerebellum—particularly the dentate nuclei and cerebellar white matter—may occur [20]. In contrast, physiological calcifications, which are part of the normal aging process of the brain, occur more frequently in the globus pallidus than in the cortex. In Fahr’s disease, early symptoms often occur, and neurological symptoms vary, but motor disorders are most common [21]. On T1WI MRI images, calcifications in the basal ganglia are characterized by increased signal intensity—they are usually extensive, bilateral, symmetrical, and involve the globus pallidus, as well as the thalamus [22]; this is analogous to the findings in our patient’s examination. Furthermore, the observed asymmetrical change in the jaw, despite its location and the patient’s gender and age, which suggests an asymptomatic bone island, requires further monitoring, preferably using CBCT (cone-beam computed tomography) [23].

In addition, our patient and her mother are carriers of a heterozygous mutation in the *TMEM67* gene. Bardet–Biedl syndrome is a heterogeneous disease; symptoms vary depending on the mutation responsible for the disease and the gene affected. Clinical variability has been described even among members of the same family [8,24]. Our patient exhibits some characteristics typical of BBS, such as obesity, hyperlipidemia, insulin resistance, polycystic ovary syndrome, hypertension, and cognitive impairment. However, her phenotype does not meet the clinical criteria for a diagnosis of BBS. Our patient meets only two major and one minor criteria suggested by Forsythe and Beales [25] (see Supplementary Table S1), while her mother meets one major and one minor criterion.

In various studies, individuals with heterozygotic variants in BBS genes were found to have specific symptoms that are characteristic of BBS. Some studies indicate that heterozygous parents of homozygous BBS patients are more prone to obesity compared to the healthy population [26,27]. Beales et al. also observed an increased incidence of hypertension and diabetes in carriers of BBS [27]. In contrast, Li et al. did not describe an increased risk of comorbidities and metabolic diseases in these patients [28]. In a study evaluating obesity without other symptoms in children, 33.3% of participants had heterozygous variants of BBS genes [29]. Recently, a newborn presenting with hydrometrocolpos and postaxial polydactyly was described to be a carrier of a single BBS mutation [30].

Therefore, the BBS carrier status, which coexists with Fahr’s disease, may be a potential contributing factor to the patient’s metabolic disorders and severe obesity; however, these conditions may be multifactorial in nature. Furthermore, it should be noted that potential structural variants were not evaluated in our patient, as intronic variants in BBS are rare and mainly affect the *BBS1*, *BBS2*, *BBS4*, *BBS7*, and *BBS16* genes [31,32,33,34].

## 4. Conclusions

In conclusion, a patient with Fahr’s disease and concurrent BBS carrier status requires multidisciplinary care, including neurological, endocrinological, ophthalmological, and dental assessments, as well as the use of available treatments, including symptomatic treatment. The presented case underscores the need to pay attention to the symptoms of overlapping genetic syndromes in a single patient, which should prompt physicians to conduct further diagnostics, also taking into account the patient’s age, as some characteristic features may develop later in life. The complexity of the phenotype observed in patients with rare diseases requires the cooperation of clinicians from various specialties and comprehensive genetic testing, including the use of NGS.

## Data Availability

The data are available to all interested persons upon request to the correspondent author.

## Supplementary Materials

The following supporting information can be downloaded at: https://www.mdpi.com/article/10.3390/genes16121406/s1. Table S1, Clinical features presented by the patient in the clinical criteria by Forsythe and Beales (2013) [25].

## Abbreviations

The following abbreviations are used in this manuscript:

ACMG	American College of Medical Genetics and Genomics
BBS	Bardet–Biedl syndrome
CBCT	cone-beam computed tomography
EMA	European Medicines Agency
FDA	Food and Drug Administration
GLP-1	glucagon-like peptide-1
NGS	next-generation sequencing
HbA1c	glycated hemoglobin
IFG	impaired fasting glycaemia
MRI	magnetic resonance imaging
OGTT	oral glucose tolerance test
PCOS	polycystic ovarian syndrome
VUS	variant of unknown significance
