# Supplementary material for: Unexpected Diagnosis of Fahr’s Disease in a Patient with Severe Obesity and a Heterozygotic Variant in the TMEM67 Gene
Source: Genes (Basel). 2025 Nov 26;16(12):1406. doi: 10.3390/genes16121406 (PMC12732503; doi:10.3390/genes16121406)
Supplement: Supplementary file 1 [file genes-16-01406-s001.zip › genes-3976336-supplementary.pdf]

Clinical sign	Patient's features
**Major features**	
Red-cone dystrophy	
Polydactyly	
Obesity	X
Genital anomalies	
Renal anomalies	
Learning difficulties	X
**Minor features**	
Speech delay	
Developmental delay	
Diabetes mellitus	
Dental anomalies	X
Congenital heart disease	
Brachydactyly/syndactyly	
Ataxia/poor coordination	
Anosmia/hyposmia	

Table S1. Clinical features presented by the patient in clinical criteria by Forsythe and Beales (2013) [25].
